# Planning and implementation of a countrywide campaign to deliver over 16 million long-lasting insecticidal nets in Mozambique

**DOI:** 10.1186/s12936-018-2406-2

**Published:** 2018-07-09

**Authors:** Jorge A. H. Arroz, Baltazar Candrinho, Sílvia Pedro, Guidion Mathe, Mariana da Silva, Sérgio Tsabete, Luis Ismael, Inês Juleca, Marta Chande, Fernando Bambo, Olinda Munguande, Sérgio Julane, Figueiredo Mussambala, Eunice Alfai, Olimpia Muianga, Hermelgildo Matsimbe, Pablo Varela, Christine Latif, Chandana Mendis, Melanie Lopez, Gagik Karapetyan, Marcy Erskine

**Affiliations:** 1 World Vision International, Maputo, Mozambique; 2 National Malaria Control Programme, Maputo, Mozambique; 3 Fundação para o Desenvolvimento da Comunidade, Maputo, Mozambique; 4 World Vision, Inc., Washington, USA; 5 Alliance for Malaria Prevention, Geneva, Switzerland

**Keywords:** Long-lasting insecticidal nets, Universal coverage campaign, Planning and implementation, Mozambique

## Abstract

**Background:**

In 2016/2017, Mozambique conducted a countrywide long-lasting insecticidal nets (LLINs) universal coverage campaign (UCC). This paper aims to describe the planning and implementation process of the campaign in Mozambique.

**Methods:**

A cross-sectional and descriptive design was used for reporting the planning and implementation process of the UCC. The UCC used a collaborative approach, involving institutional and non-institutional actors, namely: National Malaria Control Programme (NMCP), provincial and district health authorities, community members and civil society partners. A new household registration strategy based on coupons, stickers, and one LLIN per two persons as allocation criterion was implemented. The campaign was implemented in phases, allowing for continuous improvement of implementation quality by applying lessons learnt from each phase.

**Results:**

A total of 7,049,894 households were registered corresponding to a total of 31,972,626 registered persons. A total of 16,557,818 LLINs were distributed between November 2016 and December 2017, corresponding to 97% of LLINs needs based on household registration, and covering 95% of the registered households (6,708,585 households), resulting in an estimated 85% of the total Mozambican population with LLIN access.

**Conclusions:**

The collaborative planning process and strong coordination of campaign actors allowed Mozambique’s NMCP and partners to successfully carry out the first countrywide LLINs UCC in the country. The increased access to LLINs in households will likely result in increased LLIN use and a reduction of the malaria burden in the country, therefore contributing to the achievement of the 2016–2030 Global Technical Strategy for Malaria goals.

**Electronic supplementary material:**

The online version of this article (10.1186/s12936-018-2406-2) contains supplementary material, which is available to authorized users.

## Background

Despite a substantial expansion of malaria prevention and control interventions, the disease remains endemic in inter-tropic countries, with the burden heaviest in the African region, where an estimated 90% of all malaria incidence and death occurs [1, 2].

The Global Technical Strategy for Malaria 2016–2030 (GTSM) established an ambitious goal of reducing malaria mortality and morbidity by at least 90% by 2030 compared with 2015 levels [1]. The first pillar of this strategy is to ensure universal access to malaria prevention, diagnosis and treatment, and one of the harness supporting elements is innovation [1]. Proper use of long-lasting insecticidal nets (LLINs) is a preventive measure that can reduce malaria morbidity and mortality, especially in children and pregnant women [3, 4], and universal coverage LLIN campaign (UCC) is a proven health intervention for increasing population access towards achievement of this goal [5–7].

In late 2015, Mozambique piloted a new LLIN delivery strategy for UCC [8]. This pilot tested innovations, namely: coupons, stickers and one LLIN for every two persons as allocation criterion [8]. Two studies conducted in Mozambique have shown that the UCC piloted with the new delivery model has increased LLIN ownership and use, and demonstrated progress for reaching universal coverage targets [8, 9]. This new delivery model was adopted for countrywide scale-up in 2016 and 2017.

Mozambique’s National Malaria Control Programme (NMCP) set the general objective for the 2016/2017 UCC: to contribute to the reduction of malaria morbidity and mortality through LLIN universal access. Two specific objectives were set: (i) to ensure that at least 90% of registered households have sufficient LLINs (i.e., one LLIN for every two persons), and (ii) to ensure that at least 80% of LLIN owners sleep under their LLINs.

The aim of this report is to describe the planning and implementation process of the universal coverage campaign in Mozambique during 2016 and 2017. The specific objectives are: (i) to describe the planning process; (ii) to describe the implementation outcomes of the intervention; (iii) to document and share challenges and lessons learned during the campaign planning and implementation.

## Methods

### Context

**Fig. 1:**
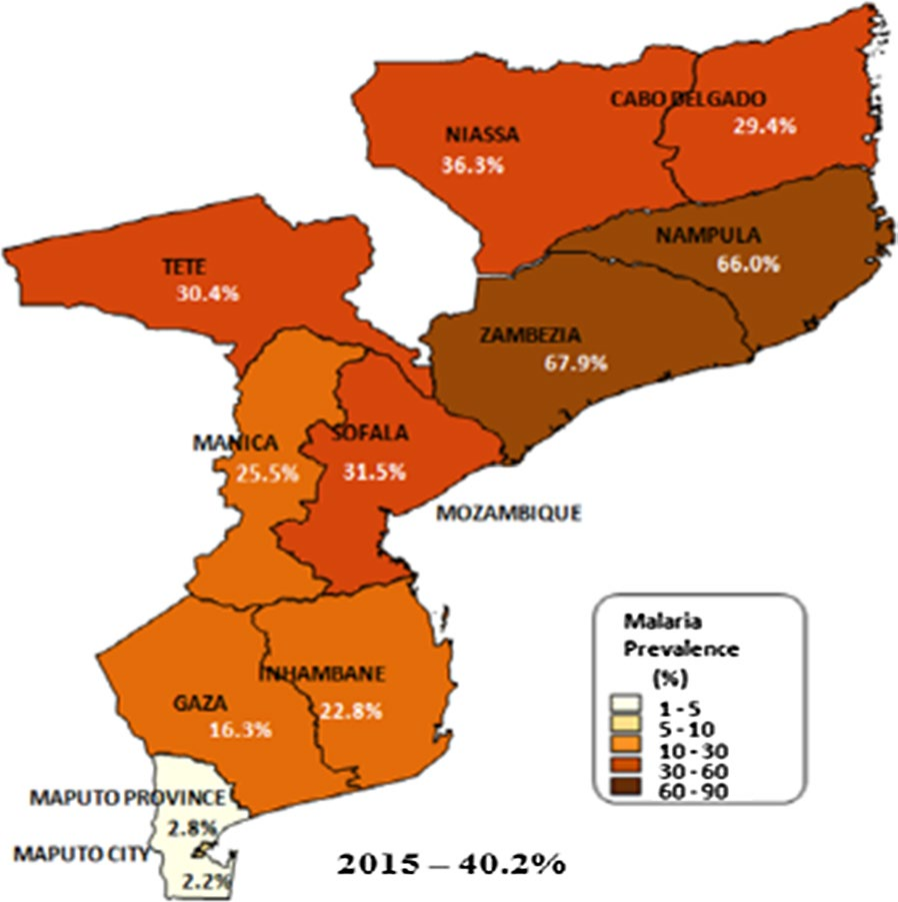
Prevalence of malaria per provinces, Mozambique 2015

In 2015, malaria prevalence in Mozambique was 40.2% [10]. The magnitude of the disease is heterogeneous, being more prevalent in central and northern regions where prevalence can reach near 70%, and less prevalent in southern regions where prevalence is less than 3% in certain provinces [10]. Five provinces have more than 30% malaria prevalence (Zambezia, Nampula, Niassa, Sofala, and Tete), four provinces have between 10 and 30% malaria prevalence (Cabo Delgado, Manica, Inhambane, and Gaza), and only two provinces have less than 3% malaria prevalence (Maputo province and Maputo city) [10] (Fig. 1).

### Design

A cross-sectional and descriptive design was used for reporting the planning and implementation process of the UCC. The description is divided into two sections: planning and implementation outcomes.

### Planning

#### Macro-planning quantification

Macro-planning was conducted in early February 2016, involving determination of the strategy for the registration and the allocation, as well as for the LLIN distribution. An initial barrier faced at the macro-planning stage was the quantification for the LLINs required to achieve universal coverage. The most recent National Population Census was conducted in 2007, almost 10 years prior to the planned campaign period. Since the National Census, Mozambique has experienced a series of natural disasters, as well as periods of political instability, both of which have led to population movement. In addition, Mozambique is experiencing increased urbanization, including in cities outside the capital and the port areas [11]. Therefore, the 2017 population projection based on the census was unreliable, and a correction factor needed to be applied to adjust population figures to more closely reflect reality. For some provinces it was possible to apply a correction factor based on the variation between population projection estimates and data collected during the household registration (HHR) phases of recent campaigns. For provinces that did not have recent campaign, a 5% correction factor was applied.

After applying this correction factor, a new at-risk population was determined and the number of LLINs required was established by dividing that population by 1.8 as recommended for LLINs quantification [12] (Table 1).

**Table 1:** Macro-quantification of long-lasting insecticidal nets for universal coverage campaign

Province	2017 population	Correction factor	2017 population with correction factor	Planned households (population/4)	Planned LLINs (pop/1.8)	Planned bales (LLINs/50)
Nampula	5,130,036	1.00	5,130,036	1,282,509	3,050,020^a^	61,000
Niassa	1,789,120	1.24	2,218,509	554,627	1,232,505	24,650
Cabo Delgado	1,952,341	1.05	2,049,958	512,490	1,138,866	22,777
North region (sub total 1)	8,871,497	1.06	9,398,503	2,349,626	5,421,390	108,428
Zambezia	5,043,120	1.14	5,749,157	1,437,289	3,193,976	63,880
Tete	2,723,010	1.05	2,859,161	714,790	1,588,423	31,768
Manica	2,071,403	1.04	2,154,259	538,565	1,196,811	23,936
Sofala	2,150,769	1.05	2,258,307	564,577	1,254,615	25,092
Central region (sub total 2)	11,988,302	1.09	13,020,884	3,255,221	7,233,824	144,676
Inhambane	1,547,850	1.05	1,625,243	406,311	902,913	18,058
Gaza	1,467,951	1.05	1,541,349	385,337	856,305	17,126
Maputo province	1,858,597	1.05	1,951,527	487,882	1,084,182	21,684
Maputo city	1,273,076	1.05	1,336,730	334,182	742,628	14,853
South region (sub total 3)	6,147,474	1.05	6,454,848	1,613,712	3,586,027	71,721
Mozambique (total)	27,007,273	1.07	28,874,234	7,218,559	16,241,241	324,825

^a^ In Nampula province, no correction factor was applied since the LLINs were already procured before the quantification decision. An additional 200,000 LLINs were added as buffer

#### Phases of campaign implementation

The campaign was planned to roll-out in phases, starting in the northern region and in provinces with the highest malaria prevalence. The roll-out plan was developed based on the following parameters: (i) provinces, (ii) number of nets, (iii) port of entry, (iv) planned date of arrival at port of entry, and (v) planned dates for distribution. Table 2 shows the detailed roll-out plan for 2017 campaign.

**Table 2:** Detailed roll-out plan 2017

	# of LLINs	Port of entry	# of bales per port of entry	# of cont. per port of entry^a^	Planned date of arrival at port	Planned date of distribution
Group 1						
Niassa	1,232,505	Nacala	24,650	56	January 2017	March 2017
Cabo Delgado	1,138,866		22,777	52		
		Total Nacala	47,427	108		
Zambezia	3,193,976	Beira	63,880	145		March 2017
Tete	1,588,423		31,768	72		April 2017
		Total Beira	95,648	217		
Total group 1	7,153,770		143,075	325		
Group 2						
Manica	1,196,811	Beira	23,936	54	April 2017	August 2017
Sofala	1,254,615		25,092	57		
		Total Beira	49,029	111		
Inhambane	902,913	Maputo	18,058	41		September 2017
Total group 2	3,354,339		67,087	152		
Group 3						
Gaza	856,305	Maputo	17,126	39	June 2017	September 2017
Maputo Province	1,084,182		21,684	49		
Maputo City	742,628		14,853	34		
Total group 3	2,683,115		53,662	122		
Grand Total	13,191,224^b^		263,824	600		

^a^ Approximate number of containers (a container can accommodate 440 bales)

^b^ Adding the 3,050,020 LLINs from Nampula province will get 16,241,244 LLINs

#### Establishment of coordination groups

The establishment of central level coordination groups was necessary for planning and for coordination of campaign actors. A National Coordination Group (NCG) was created which, with the support of the four National Technical Sub-Groups (NTSG, described below), coordinated the planning and implementation of the UCC. The NCG was led by the NMCP manager and assisted by the Director of the Malaria Project funded by the Global Fund—World Vision Mozambique. Other members of this group included representatives of international and national partners that cooperate with NMCP, such as the US President’s Malaria Initiative (PMI) and the World Health Organization (WHO).

**Fig. 2:**
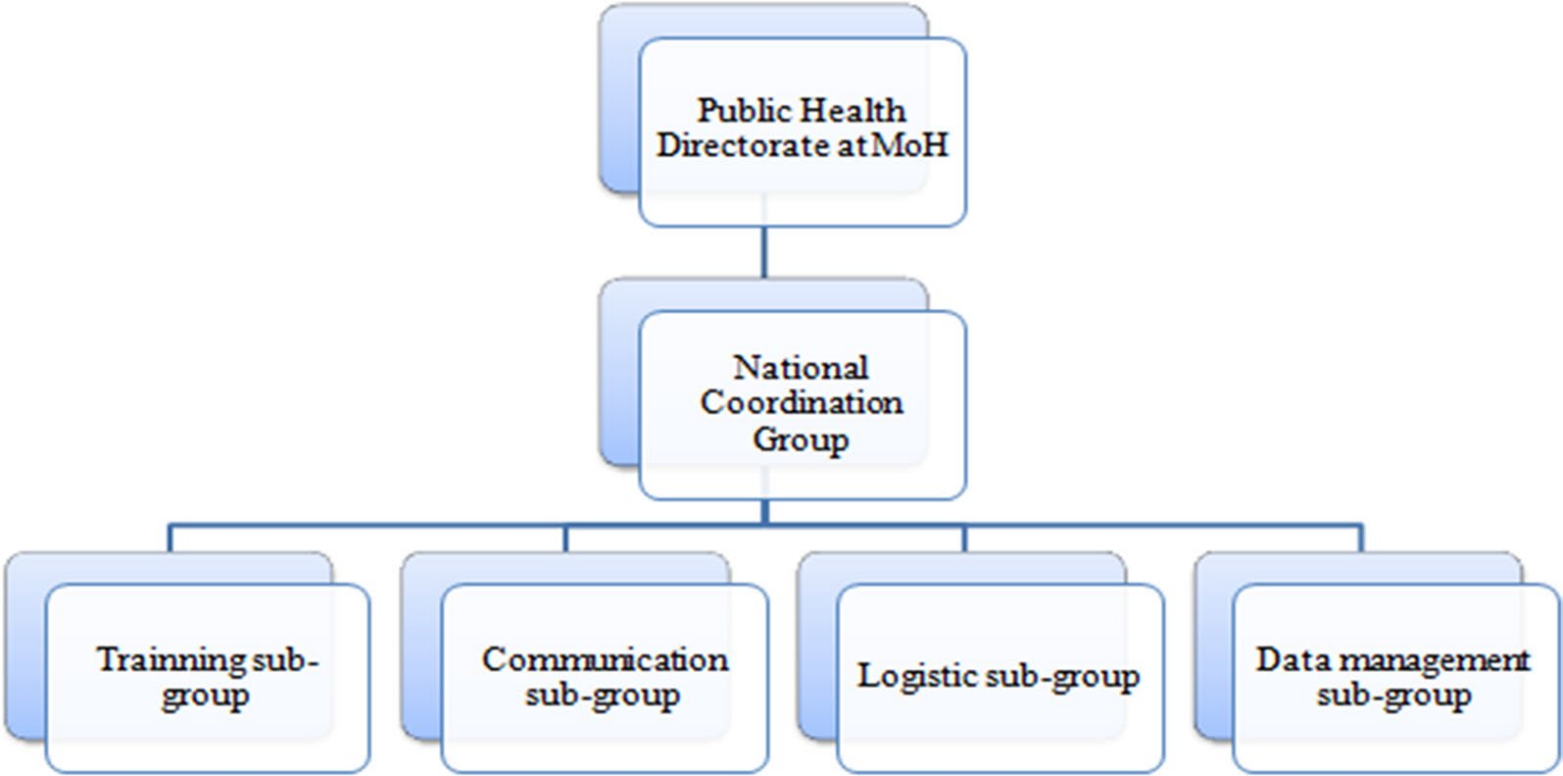
National Coordination Group and Technical Sub-Groups

Four NTSG were also created in order to give technical support to the NCG in the following areas: training; communication; logistics; and data management. These NTSG provided technical inputs to the decision-making process of the NCG, while the NCG reported to a higher level of decision-making at the Ministry of Health (MoH), the Public Health Directorate (Fig. 2). The Public Health Directorate was responsible for providing necessary information to the Minister of Health, who would disseminate UCC updated information to all Ministries. This structure contributed to multi-level engagement, advocacy, and mobilization.

**Fig. 3:**
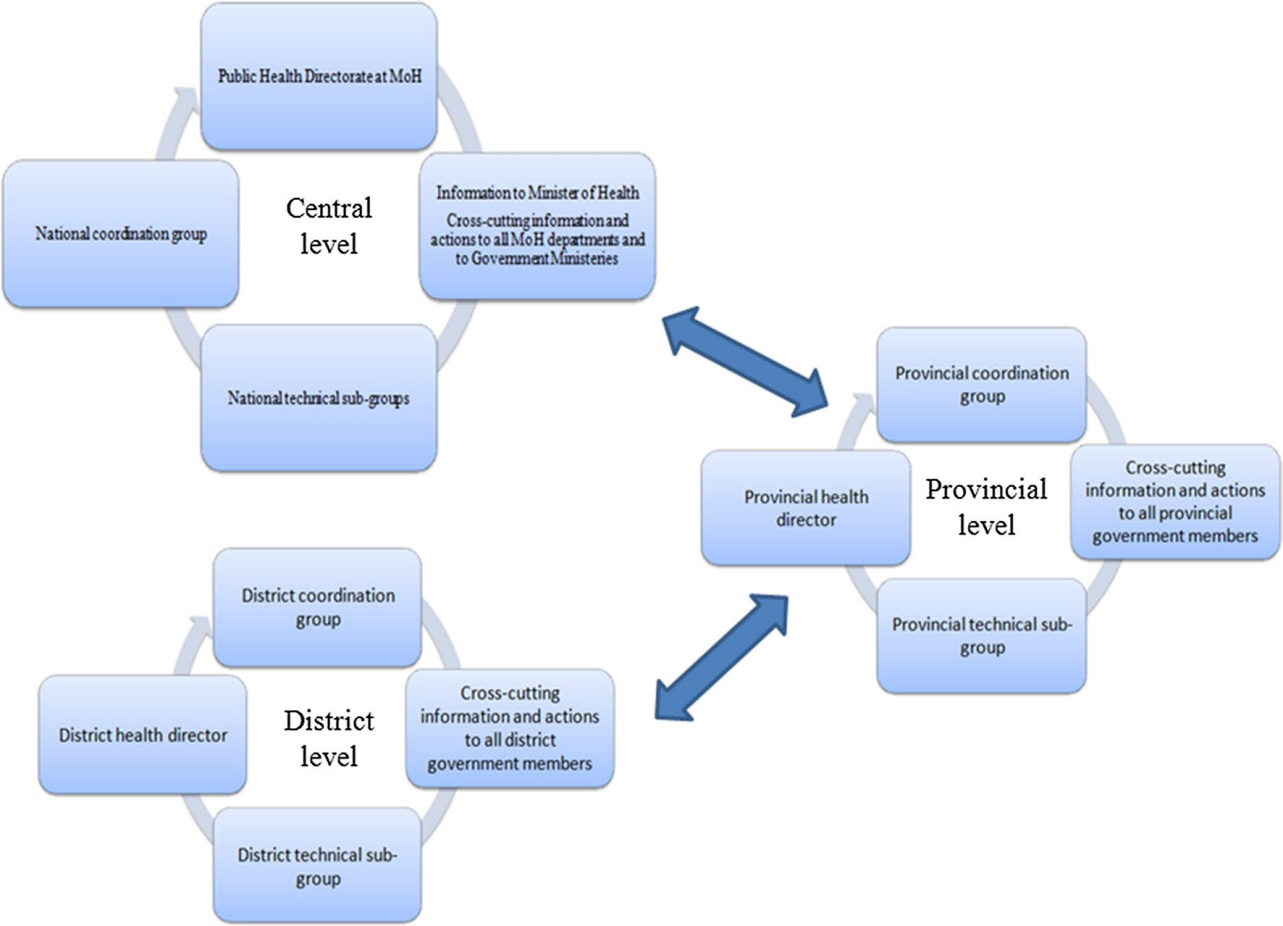
Central, provincial and district level coordination groups and information flow

At provincial and district level, similar structures were created: provincial/district coordination groups, and provincial/district technical sub-groups. The provincial coordination group (PCG) was a political engagement and advocacy group, composed of government members from different provincial sectors: health, agriculture, fishery, education, police, and others. The PCG was chaired by the provincial Governor and coordination meetings were organized on a regular basis by the Governor. The district coordination group (DCG) had a similar role and composition, led by the district administrator (Fig. 3).

The provincial technical sub-group (PTSG) was a technical group that coordinated the campaign implementation, and was composed of the Provincial Medical Chief, Head of Public Health Department, Malaria Focal Point, Community Involvement Focal Point, Implementation Partner Coordinator, and other provincial health technical staff. The PTSG held regular coordination meetings on a weekly basis. The district technical sub-group (DTSG) had similar role and composition (Fig. 3). The information was shared from district to provincial to central level, in a two-way sense (information submission and feedback) as shown in Fig. 3.

#### Campaign tools

The following main campaign tools were developed and used during 2016/2017 UCC:

- Micro-planning tool: Used during micro-planning phase in order to create a positioning plan. The micro-planning tool has been previously described and shared elsewhere [8];
- Household registration tools: Coupons, stickers, and household registration sheets. Coupons and stickers have been previously described and shared elsewhere [8]. The household registration sheet is available as Additional file 1: Appendix S1;
- Distribution phase tools: Benefited household tally sheet and distributed LLINs tally sheet. The tools are available as Additional file 2: Appendix S2 and Additional file 3: Appendix S3;
- Logistics and LLIN tracking tools: Warehouse stock form and waybills for use during LLIN transport, used to ensure high accountability for the LLINs through the supply chain based on the provincial transport plan. The tools are available as Additional file 4: Appendix S4 and Additional file 5: Appendix S5;
- Operational payments plan: An operational payments plan was developed to map and identify payment methods according to different involved actors. Three payment methods were considered: bank transfer, mobile money and cash-on-hand. An example of detailed operational payments plan is available as Additional file 6: Appendix S6.

#### Advocacy, engagement meetings and mobilization

In each province, an advocacy and engagement meeting preceded (1 month ahead) the micro-planning workshop. High level representatives of the government from each district and province participated in the meetings, which were facilitated by technical staff from NMCP and Civil Society Partners, to explain the campaign purpose and strategies, and discuss ways that these influential people could support the campaign planning and implementation. Following the provincial engagement, journalists from each district and province were trained (a total of 134 journalists) for mobilization around campaign implementation. Radio and television spots were created and broadcast during household registration and distribution phases to mobilize the population to participate in the campaign and receive LLINs.

Additionally, mobilization meetings took place at each district involving the district government and other stakeholders including: political parties, associations of traditional healers, community leaders, influential members in the district (local businessmen, private sector service providers) and community radio coordinators.

Advocacy materials were created to support mobilization of the population and ensure consistency in information received and disseminated. Materials included: guides for leaders, journalists and influential individuals; t-shirts with the image of a LLIN hanging and a family sleeping under the LLIN; education pamphlets on LLIN use and care; and a job aid for use during the household registration. A free central level telephone hotline functioned 24 h a day/7 days a week with the purpose of clarifying doubts about the campaign and responding to questions regarding malaria or the LLINs.

#### Trainings

Trainings were organized in a cascade manner at central, provincial and district levels. Three central-level training of trainers (ToT) sessions were carried out, allowing development of the necessary tools for implementation, ensuring lessons learned and modifications were incorporated in each subsequent campaign phase and creating equal understanding among central-level staff regarding the campaign implementation strategies. In each province, separate micro-planning workshops and TOT sessions for implementation of the campaign took place to train the provincial and district coordination groups. The cascade trainings have been previously described elsewhere [8].

#### Household registration planning and implementation

Household registration was conducted by community volunteers whom had at least a grade 7 level of education. Each household registrar had to register between 20 and 30 households per day over a period of 7 days. The household registration strategy was based on the coupon-sticker demand effect observed during the pilot study, i.e., following the household registration, households received vouchers for LLINs in the form of coupons that they redeemed at a pre-specified location [8, 9].

#### LLIN allocation

During implementation in Nampula province, where the revised strategy was first piloted at scale, no maximum number of LLINs per household was established. Over the course of the provincial pilot, a maximum of five LLINs per household was established for some districts province due to observed inflation of number of household members [8]. Following the provincial pilot, the NCG took a decision to set a maximum of four LLINs per household for the remaining provinces. In the last phase of the campaign, which targeted Maputo province and Maputo city, a maximum of three LLINs per household was established due to some apprehensiveness of LLIN shortage related to movement of nets procured for provinces in the southern region to the central and northern regions to fill gaps in LLINs following household registration (Table 6 in “Results” section). As a consequence of these movements, five out of seven districts of Maputo city were not covered during the LLIN distribution.

#### Rapid monitoring of household registration

Rapid monitoring of household registration was carried out using Lot Quality Assurance Sampling (LQAS). Each district to be assessed had a team of seven monitors, with a leader for each team. The steps and results of the rapid monitoring are available as Additional file 7: Appendix S7.

#### Distribution team composition and roles

The distribution teams involved in the LLIN distribution phase (planned for 5 continuous days, with maximum of 2 days extra) were composed of five fixed elements and three non-fixed elements. The five fixed elements were: one team leader, one mobilizer, one coupon receptor, and two LLIN distributors. The three non-fixed elements were local community assistants, recruited at the distribution point by the team leader to help with organizing the queues, moving bales from vehicles to distribution points and distributors, opening the LLIN bales, cleaning up the distribution point, or performing other tasks as requested by the team leader.

### Implementation outcomes

The following two categories of indicators are herein reported:

#### Process indicators

Number of household registrars involved; number of distribution teams (DTs); LLIN needs after household registration variation (%) calculated using the following formula: (LLINs needs after household registration − macro-planned LLINs): macro-planned LLINs × 100; registered population variation (%) calculated using the following formula: (registered population − macro-planned population):macro-planned population × 100; and registered households variation (%) calculated using the following formula: (registered households − macro-planned households): macro-planned households × 100.

#### Performance indicators

Ratio of households (HH) to household registrars (HHR) by day, calculated by dividing registered households by number of registrars and dividing by 7 registration days; average number of households served per day per distribution team, calculated by dividing registered households by number of DTs and dividing by 5 distribution days; average number of LLINs distributed per day per DT, calculated by dividing LLINs distributed by number of DTs and dividing by 5 distribution days; percentage of LLINs distributed, calculated using the following formula: distributed LLINs: LLINs needs after household registration × 100; percentage of households served with LLINs, calculated using the following formula: benefited households: registered households × 100; and estimated population access to LLINs (calculated by multiplying the number of distributed LLINs by 1.6 and dividing by target population [13]).

## Results

The 2016/2017 universal coverage campaign was launched on 3rd November 2016 in Niassa province by the President of the Republic of Mozambique. The campaign ended on 6th December 2017 in Maputo province.

A total of 55,875 household registrars were trained and involved, which gives an average ratio of 18 households being registered per household registrar, per day (range 16–21). A total of 5897 distribution teams were trained and involved in the campaign, corresponding to an average of 239 benefited households per day per distribution team (range 191–474), and an average of 562 distributed LLINs per day per distribution team (range 496–1146) (Table 3).

**Table 3:** Household registrars, distribution teams, and their ratios for each province

	HH registrars	Ratio HHs:HHR/day	Distribution teams (DT)	Average benefited HHs per day per DT	Average distributed LLINs per day per DT
Nampula	9216	21	1249	220	566
Niassa	4300	16	494	200	506
Cabo Delgado	4732	18	253	474	1146
North region (sub total 1)	18,248	19	1996	247	625
Zambezia	12,132	17	1286	226	500
Tete	5861	18	599	244	553
Manica	4164	20	425	280	610
Sofala	4868	16	577	191	498
Central region (sub total 2)	27,025	18	2887	231	526
Inhambane	3150	17	330	227	513
Gaza	3206	17	341	225	496
Maputo province	4093	17	332	290	610
Maputo city	151	21	11	289	557
South region (sub total 3)	10,600	17	1014	247	537
Mozambique (total)	55,873	18	5897	239	562

### Registered households

A total of 7,049,894 households were registered (less 2.3% from macro-planning) corresponding to a total of 31,972,626 persons (11% more from macro-planning figures). This figure corresponds to a need of 17,152,147 LLINs (6% more than macro-planning) (Table 4). It is important to note that all seven districts of Maputo city were considered in the macro-planning process; however, due to shortage of LLINs between macro-planning and actual needs after household registration in the central and north regions, five urban districts of Maputo city were not targeted for LLINs distribution, with only two districts receiving LLINs. This explains why the variation between registered households and planned households is − 97% (Table 4).

**Table 4:** Registered population, households, and LLINs needs after household registration

Province	Registered population	Variation (%)	Registered households	Variation (%)	LLINs needs after household registration	Variation (%)
Nampula	7,038,427	37.2	1,373,002	7.1	3,826,592	25.5
Niassa	2,379,284	7.2	493,628	(11.0)	1,282,914	4.1
Cabo Delgado	2,710,600	32.2	599,492	17.0	1,469,623	29.0
North region (sub total 1)	12,128,311	29.0	2,466,122	5.0	6,579,129	21.4
Zambezia	6,541,511	13.8	1,455,063	1.2	3,285,654	2.9
Tete	3,182,359	11.3	731,218	2.3	1,687,539	6.2
Manica	2,261,066	5.0	595,700	10.6	1,354,132	13.1
Sofala	2,704,880	19.8	551,534	(2.3)	1,448,876	15.5
Central region (sub total 2)	14,689,816	12.8	3,333,515	2.4	7,776,201	7.5
Inhambane	1,547,480	(4.8)	375,291	(7.6)	874,857	(3.1)
Gaza	1,555,874	0.9	383,511	(0.5)	855,074	(0.1)
Maputo province	2,015,349	3.3	481,920	(1.2)	1,048,021	(3.3)
Maputo city	35,796	(97.3)	9535	(97.1)	18,865	(97.5)
South region (sub total 3)	5,154,499	(20.1)	1,250,257	(22.5)	2,796,817	(22.0)
Mozambique (total)	31,972,626	10.7	7,049,894	(2.3)	17,152,147	5.6

### Delivered LLINs, households served and estimated population access

A total of 16,557,818 LLINs were distributed from November 2016 through December 2017, which corresponds to 96.5% of the LLIN needs after household registration (Table 5). If the percentage of distributed LLINs is based on the macro-planning figures, administrative coverage would be 102% (16,557,818/16,241,241). It should be noted that during the roll out of the campaign by phases, gaps in LLINs based on household registration were filled by moving LLINs from one province to another (Table 6). The LLINs distributed benefited 95.2% (6,708,585/7,049,894) households based on household registration data (Table 5).

**Table 5:** Distributed LLINs, households served and estimated LLIN access indicator

Province	Distributed LLINs	%	Benefited households	%	Population with LLIN access (%)
Nampula	3,536,839	92.4	1,353,827	98.6	82.4
Niassa	1,248,797	97.3	469,364	95.1	86.1
Cabo Delgado	1,450,317	98.7	599,576	100.0	87.7
North region (sub total 1)	6,235,953	94.8	2,422,767	98.2	84.3
Zambezia	3,212,615	97.8	1,327,234	91.2	80.5
Tete	1,654,854	98.1	678,316	92.8	85.3
Manica	1,295,386	95.7	533,668	89.6	94.0
Sofala	1,436,876	99.2	532,462	96.5	87.1
Central region (sub total 2)	7,599,731	97.7	3,071,680	92.1	84.8
Inhambane	846,090	96.7	351,678	93.7	89.7
Gaza	845,089	98.8	371,285	96.8	89.1
Maputo province	1,012,571	96.6	482,331	100.1^a^	82.4
Maputo city	18,384	97.5	8844	92.8	84.2
South region (sub total 3)	2,722,134	97.3	1,214,138	97.1	86.6
Mozambique (total)	16,557,818	96.5	6,708,585	95.2	84.9

^a^ 411 households in Maputo province received LLINs despite not having coupons

**Table 6:** Lateral movements of LLINs from one province to another

From which province	Source	To which province	Purpose	Number of LLINs
Maputo Province	UCC	Sofala	UCC	637,985
		Tete		47,000
		Zambezia		52,242
Sofala	UCC	Manica	UCC	175,900
		Zambezia		233,450
Nampula	ANC	Niassa	UCC	165,000
		Cabo Delgado		72,000
		Nampula		768,950
Maputo City	UCC	Maputo Province	UCC	726,084
Zambezia	UCC	Cabo Delgado	UCC	229,750
Niassa^a^	UCC	Nampula	ANC	158,160
Inhambane^b^	UCC	Maputo City	UCC	70,000
		Tete		17,000
Total				3,353,521

^a^ Devolution of borrow LLINs from ANC

^b^ LLINs from Bill and Melinda Gates Foundation

### LLIN lateral movements

Table 6 summarizes the lateral movements of LLINs from one province to another during the roll out of the campaign and disaggregates the total quantity of LLINs moved by source (campaign versus antenatal care service LLINs). Overall 3,353,521 LLINs were moved from one province to another. Of these, 1,005,950 (31.5%) LLINs were from antenatal care service (ANC). An additional amount of 100,000 LLINs were donated by the Bill and Melinda Gates Foundation to support LLIN distribution in Inhambane province. These donated LLINs were received and distributed in the country at the end of November 2017.

## Discussion

The first countrywide UCC ever conducted in Mozambique resulted in a distribution of 97% of LLIN needs based on household registration data, covering 95% of the registered households. This results in an estimated 85% of the targeted population having LLIN access in Mozambique. The total financial cost of implementation from providers perspective was US$ 20,280,750, which gives an average cost per delivered LLIN of US$ 1.22.

During the household registration, results from rapid monitoring showed that none of the districts met the criterion for re-doing household registration, and the overall number of fails during the household registration for the three main criteria amounted to less than 3% of households surveyed (Additional file 7: Appendix S7). However, the number of fails for household registrars providing an explanation on how to correctly use LLINs was higher (around 12% of households), and nearly 8% of households during registration phase did not know where the distribution point was located (Additional file 7: Appendix S7).

These results have some implications. Once households have been registered and LLINs pre-positioned for distribution, they should then go to their respective distribution points and exchange their coupons for LLINs. The number of LLINs transported to each distribution point is based on the results of the household registration, which provides the number of nets needed to reach all households around the distribution point. A household member not knowing the location of their distribution center can lead to a number of outcomes:

- The household does not exchange their coupon for LLINs and thus is not covered, affecting achievement of the campaign outcomes;
- The household attends an incorrect distribution point and is told to go a different location for their LLIN, incurring time and potential transport costs to receive the LLIN and potential non-reception of LLINs;
- Over-crowding in some of the distribution points, therefore resulting in long queues and dissatisfaction of some household members when there are insufficient LLINs;
- Fewer people attending distribution centers than planned, resulting in a surplus of LLINs while people are being served at a different distribution point that will have a shortage of LLINs;
- Lateral movement of LLINs in the middle of the distribution, with risks that tracking tools are not filled out correctly as situations are urgent.

Fortunately, the number of households that did not know the distribution point was not excessive.

Despite some household members not receiving a proper explanation on how to correctly use LLINs, the number of households was also not high. In fact, household registrars have a dual role—to register and mobilize—but the quality of the two roles during implementation is not always equal. Training plays an important part in what the household registrar-mobilizer will consider to be the more important part of their role, based on both the emphasis put on various topics by the trainers, but also in the time spent on topics over the course of the training.

## Operational challenges

The first challenge was related to the planning process to coordinate and roll out the campaign. The major challenge in this phase was the need to re-create all the necessary tools for running the campaign once the new strategy was formally adopted, as well as to increase accountability for the LLINs. This included: standard budget, micro-planning tool, logistic plan of action and supporting tools for LLIN accountability, transport plan, household registrar forms, coupons and stickers, and tally sheets for household served and LLINs distributed.

The second challenge was with the micro-planning process, ensuring that the district level stakeholders remained strongly engaged and highly active during all the campaign phases. Real time communication through various channels (WhatsApp, e-mail, telephone call) enabled the central team to coordinate with all provinces and districts and provide necessary technical support, even when not physically present.

At the early stage of the campaign implementation (Nampula province), the absence of a maximum number of LLINs per household and a lack of coordination and planning between the implementing actors at provincial level was a challenge, leading to constraints, including slow recognition and resolution of problems [8].

Lessons learnt from Nampula province guided improvements for the next provinces: coordination was reinforced (strong engagement of provincial health authorities, high level political engagement at all levels); coupon production was centralized to ensure that the suppliers followed the standards established; and a maximum of four LLINs per household was established.

The third set of operational challenges were related to logistics and can be divided into two main aspects: materials and payment of campaign actors.

### Challenges with materials

Challenges with materials were mostly related to quantification, given the discrepancy between macro-planning (based on population projections) and micro-planning (based on information collected in each district) figures. Despite early quantification for materials and including a 20% security stock, substantial population changes after micro-planning affected the needs for materials. Given these problems, additional materials had to be ordered with short notice and delivery time, which at times delayed the roll out of campaign activities.

### Challenges with payment of campaign actors

Campaigns involve a significant number of actors that need to be paid and there is a strong push from funding partners to move away from cash payments given the high risks (e.g. transport of money, payment in non-secure locations, inability to control communication about payments leading to many people being informed).

The majority of the 55,873 household registrars (corresponding to a labour payment allowance of more than US$ 2 million for household registration) did not have formal bank accounts and their location in remote areas of districts made cash payments challenging. The banking system in Mozambique is weak, concentrated in very few economically important districts and in urban areas. In terms of payments by mobile phone, the availability (or penetration rate) of cell phones among households in Mozambique is still low, covering 45.6% of households in rural settings [14].

Based on a socio-economic and context analysis, a mix of three payment methods (bank transfers, mobile money payment, and cash-on-hand) was adopted in order to reduce cash-on-hand payments.

The experience of implementing these three methods demonstrated some challenges, particularly related to bank and mobile money payments:

- Errors in some beneficiaries’ bank account information created delays for fund transfers when they were ordered;
- Accounts for beneficiaries from different banks than the sender’s bank created delays in payments, since with different banks, the providers bank transfer order becomes available in the beneficiaries’ account only after 72 h;
- Low mobile money agent coverage made this payment method unsuitable for the majority of beneficiaries in remote rural areas;
- In areas where mobile money agent coverage was high, the mobile money agents’ liquidity was limited;
- Selected mobile money operators faced operational limitations, which in turn limited the mobile money payment option.

With these constraints, the cash-on-hand payment had to be used often (despite risks related to theft, security of those doing the payments at community level, and financial reporting). Even with this payment method, long distances and shortages of payment personnel created delays, which resulted in some frustration for the beneficiaries. At some point, the banks also became part of the problem given disruption in liquidity of funds.

These constraints have been translated into lessons learned:

- Payment planning process should be prepared early, involving discussions with banks, mobile money operators and their field agents to ensure sufficient liquidity;
- Collecting beneficiaries’ bank account information and mobile phone numbers well in advance of the payment period is crucial to allow time for a crosscheck and confirmation of the accounts and mobile phone numbers and make adjustments to avoid payment delays;
- Mobile money payment should only be considered in urban or rural areas with a strong phone network and a good ratio of mobile money agents to beneficiaries. Mapping these areas is crucial for a successful mobile money payment;
- Micro-planning information should be used to develop a payment plan for each district based on resources available across the three payment options;
- It is of paramount importance to explain to the beneficiaries in advance that some payment delays may be experienced and to provide a window of time between the end of the activity and payments.

Last but not least, the fourth challenge was central team burnout. The same central team was involved in the planning, coordination, implementation, and problem-solving of the campaign for more than 1 year. Recruitment of the highest performing technical personnel from provinces completing campaigns in order to have them support provinces in the subsequent campaign phases is a recommended strategy to avoid burnout. Despite these challenges, the campaign was successfully implemented and contributed to increasing household ownership and population access to LLINs, as well as their use, making a significant contribution to the reduction of the malaria burden in the country.

## Conclusions

Joint planning and coordination of NMCP and Civil Society Partners made it possible to successfully carry out the first countrywide LLIN universal coverage campaign in Mozambique. High political commitment, engagement and support were essential factors for success. The LLIN universal coverage campaign in Mozambique, distributing over 16 million nets, has resulted in increased LLIN ownership and use, with potential implications for the reduction of the malaria burden, therefore contributing to the achievement of the Global Technical Strategy for Malaria 2016–2030 goals. The authors strongly recommend early programme, financial, and logistics planning for future campaigns, ensuring lessons learned from this campaign are taken into account to improve implementation quality.

## Additional files

**Additional file 1: Appendix 1.** Household registration form.

**Additional file 2: Appendix 2.** Benefited household tally sheet.

**Additional file 3: Appendix 3.** Distributed LLINs tally sheet.

**Additional file 4: Appendix 4.** Warehouse stock form.

**Additional file 5: Appendix 5.** LLINs tracking waybills.

**Additional file 6: Appendix 6.** Operational payments plan.

**Additional file 7: Appendix 7.** Results of the household registration rapid monitoring.

## Abbreviations

ANC: antenatal care service
DCG: district coordination group
DT: distribution teams
DTSG: district technical sub-group
HHR: household registrars/household registration
HHs: households
LLINs: long-lasting insecticidal nets
LQAS: Lot Quality Assurance Sampling
MoH: Ministry of Health
NCG: National Coordination Group
NMCP: National Malaria Control Programme
NTSG: National Technical Sub-Group
PCG: provincial coordination group
PMI: President Malaria Initiative
PTSG: provincial technical sub-group
UCC: universal coverage campaign
WHO: World Health Organization
