# Supplementary figures and images for: Planning and implementation of a countrywide campaign to deliver over 16 million long-lasting insecticidal nets in Mozambique
Source: Malar J. 2018 Jul 9;17:254. doi: 10.1186/s12936-018-2406-2 (PMC6038318; doi:10.1186/s12936-018-2406-2)

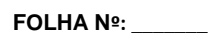

### FICHA DE REGISTO DOS AGREGADOS FAMILIARES

DATA:           /       /

[illegible]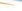

|          |                          |
|----------|--------------------------|
| Pagina # | Assinatura do supervisor |
|----------|--------------------------|

Supplement: Supplementary file 1 — Additional file 1: Appendix 1. Household registration form. [file 12936_2018_2406_MOESM1_ESM.pdf]
