# Supplementary material for: Planning and implementation of a countrywide campaign to deliver over 16 million long-lasting insecticidal nets in Mozambique
Source: Malar J. 2018 Jul 9;17:254. doi: 10.1186/s12936-018-2406-2 (PMC6038318; doi:10.1186/s12936-018-2406-2)
Supplement: Supplementary file 2 — Additional file 2: Appendix 2. Benefited household tally sheet. [file 12936_2018_2406_MOESM2_ESM.pdf]

RURAL

MINISTERIO DE SAUDE
FICHA DE AGREGADOS FAMILIARES BENEFICIADOS

PROVÍNCIA ___________________________ **Distrito** ______________________ **Posto Administrativo:** ___________________

(NB: Em cada dia de distribuicao usar uma ficha nova de registo)

Localidade: ______________________ Povoado/Comunidade/Bairro ___________________________

Posto de Distribuição: _________________ **Data** __/ __/ ______

A SER PEENCHIDO PELO RECEPTOR DE SENHAS

AGREGADOS FAMILIARES BENEFICIADOS										TOTAL	
00000	00000	00000	00000	00000	00000	00000	00000	00000	00000	50	
00000	00000	00000	00000	00000	00000	00000	00000	00000	00000	100	
00000	00000	00000	00000	00000	00000	00000	00000	00000	00000	150	
00000	00000	00000	00000	00000	00000	00000	00000	00000	00000	200	
00000	00000	00000	00000	00000	00000	00000	00000	00000	00000	250	
00000	00000	00000	00000	00000	00000	00000	00000	00000	00000	300	
00000	00000	00000	00000	00000	00000	00000	00000	00000	00000	350	
00000	00000	00000	00000	00000	00000	00000	00000	00000	00000	400	
00000	00000	00000	00000	00000	00000	00000	00000	00000	00000	450	
00000	00000	00000	00000	00000	00000	00000	00000	00000	00000	500	
00000	00000	00000	00000	00000	00000	00000	00000	00000	00000	550	
00000	00000	00000	00000	00000	00000	00000	00000	00000	00000	600	
00000	00000	00000	00000	00000	00000	00000	00000	00000	00000	650	
00000	00000	00000	00000	00000	00000	00000	00000	00000	00000	700	
00000	00000	00000	00000	00000	00000	00000	00000	00000	00000	750	
00000	00000	00000	00000	00000	00000	00000	00000	00000	00000	800	
00000	00000	00000	00000	00000	00000	00000	00000	00000	00000	850	
00000	00000	00000	00000	00000	00000	00000	00000	00000	00000	900	
00000	00000	00000	00000	00000	00000	00000	00000	00000	00000	950	
00000	00000	00000	00000	00000	00000	00000	00000	00000	00000	1,000	
**TOTAL de Agregados Familiares Beneficiados**											

Nome do chefe da equipa do PD _______________________ **Assinatura** ______________ **Data** ___/___/ ____
Observações:

____
