# Supplementary material for: Planning and implementation of a countrywide campaign to deliver over 16 million long-lasting insecticidal nets in Mozambique
Source: Malar J. 2018 Jul 9;17:254. doi: 10.1186/s12936-018-2406-2 (PMC6038318; doi:10.1186/s12936-018-2406-2)
Supplement: Supplementary file 3 — Additional file 3: Appendix 3. Distributed LLINs tally sheet. [file 12936_2018_2406_MOESM3_ESM.pdf]

RURAL

## MINISTÉRIO DE SAÚDE
### FICHA DE DISTRIBUIÇÃO DE REDES MOSQUITEIRAS

**PROVÍNCIA** __________________________ **Distrito** _____________________ **Posto Administrativo:** __________________

*(NB: Em cada dia de distribuicao usar uma ficha nova de registo)*

Localidade: _____________________ Povoado/Comunidade/Bairro __________________________

**Posto de Distribuição:** __________________________ **Data:** ____ / ____ /______

**A SER PEENCHIDO PELO DISTRIBUIDOR**

| REDES MOSQUITEIRAS DISTRIBUÍDAS | | | | | | | | | | TOTAL | |
|---|---|---|---|---|---|---|---|---|---|---|---|
| 00000 | 00000 | 00000 | 00000 | 00000 | 00000 | 00000 | 00000 | 00000 | 00000 | 50 | |
| 00000 | 00000 | 00000 | 00000 | 00000 | 00000 | 00000 | 00000 | 00000 | 00000 | 100 | |
| 00000 | 00000 | 00000 | 00000 | 00000 | 00000 | 00000 | 00000 | 00000 | 00000 | 150 | |
| 00000 | 00000 | 00000 | 00000 | 00000 | 00000 | 00000 | 00000 | 00000 | 00000 | 200 | |
| 00000 | 00000 | 00000 | 00000 | 00000 | 00000 | 00000 | 00000 | 00000 | 00000 | 250 | |
| 00000 | 00000 | 00000 | 00000 | 00000 | 00000 | 00000 | 00000 | 00000 | 00000 | 300 | |
| 00000 | 00000 | 00000 | 00000 | 00000 | 00000 | 00000 | 00000 | 00000 | 00000 | 350 | |
| 00000 | 00000 | 00000 | 00000 | 00000 | 00000 | 00000 | 00000 | 00000 | 00000 | 400 | |
| 00000 | 00000 | 00000 | 00000 | 00000 | 00000 | 00000 | 00000 | 00000 | 00000 | 450 | |
| 00000 | 00000 | 00000 | 00000 | 00000 | 00000 | 00000 | 00000 | 00000 | 00000 | 500 | |
| 00000 | 00000 | 00000 | 00000 | 00000 | 00000 | 00000 | 00000 | 00000 | 00000 | 550 | |
| 00000 | 00000 | 00000 | 00000 | 00000 | 00000 | 00000 | 00000 | 00000 | 00000 | 600 | |
| 00000 | 00000 | 00000 | 00000 | 00000 | 00000 | 00000 | 00000 | 00000 | 00000 | 650 | |
| 00000 | 00000 | 00000 | 00000 | 00000 | 00000 | 00000 | 00000 | 00000 | 00000 | 700 | |
| 00000 | 00000 | 00000 | 00000 | 00000 | 00000 | 00000 | 00000 | 00000 | 00000 | 750 | |
| 00000 | 00000 | 00000 | 00000 | 00000 | 00000 | 00000 | 00000 | 00000 | 00000 | 800 | |
| 00000 | 00000 | 00000 | 00000 | 00000 | 00000 | 00000 | 00000 | 00000 | 00000 | 850 | |
| 00000 | 00000 | 00000 | 00000 | 00000 | 00000 | 00000 | 00000 | 00000 | 00000 | 900 | |
| 00000 | 00000 | 00000 | 00000 | 00000 | 00000 | 00000 | 00000 | 00000 | 00000 | 950 | |
| 00000 | 00000 | 00000 | 00000 | 00000 | 00000 | 00000 | 00000 | 00000 | 00000 | 1,000 | |
| TOTAL de REMILDs Distribuídas | | | | | | | | | | | |

**A: Quantidade de Redes Mosquiteiras recebida do armazém do PD no início do dia** ______________________________________

**B: Quantidade adicional de Redes Mosquiteiras recebida do armazém do PD** ___________________________________________

**C: Redes Mosquiteiras distribuida hoje** _______________________________________________________________________

**D: Quantidade de Redes Mosquiteiras que sobraram no fim do dia** __________________ **(devolvidos ao armazém do PD)**

**Nome do chefe da equipa do PD** ________________________**Assinatura** __________________ **Data** ____/___/ ___

**Observações:**
