# Supplementary material for: Planning and implementation of a countrywide campaign to deliver over 16 million long-lasting insecticidal nets in Mozambique
Source: Malar J. 2018 Jul 9;17:254. doi: 10.1186/s12936-018-2406-2 (PMC6038318; doi:10.1186/s12936-018-2406-2)
Supplement: Supplementary file 4 — Additional file 4: Appendix 4. Warehouse stock form. [file 12936_2018_2406_MOESM4_ESM.pdf]

Ministério de Saúde

Programa Nacional de Controlo da Malária

CAMPANHA DE DISTRIBUIÇÃO DE REDES MOSQUITEIRAS PARA A COBERTURA UNIVERSAL

FICHA DE STOCK DE ARMAZÉM

Pagina: ____/____

Província de: ___________________________ **Distrito de:** _____________________________ **Localidade de:** __________________________

Nome do Responsável do Armazém: _____________________________________

Nota: use esferográficas de cores diferentes para diferenciar os movimentos de entradas e de saídas (Vermelho para entradas e azul/preta para saídas)

Nr.	Data	Proveniência (Coluna a ser preenchida só para entradas)	Destino (coluna a ser preenchida só para saídas)	Nr. da guia de remessa	Nr. matrícula ou tipo de transporte	Quantidade de redes indicadas na guia de remessa	Quantidade de redes recebidas (Coluna a ser preenchida só para entradas)	Quantidade de redes enviadas (coluna a ser preenchida só para saídas)	Stock			Comentários / Nome e apelido
									Nr. total de REMILDs	Nr. de Redes Mosquiteiras Fora do Fardo	Nr. Fardos não abertos	
									A = B + (C X 50)	B	C	

Total de entradas	Total de saídas

Stock de Fardos a transitar:	
Stock de REMILDs avulsas a transitar:	
