# Supplementary material for: Planning and implementation of a countrywide campaign to deliver over 16 million long-lasting insecticidal nets in Mozambique
Source: Malar J. 2018 Jul 9;17:254. doi: 10.1186/s12936-018-2406-2 (PMC6038318; doi:10.1186/s12936-018-2406-2)
Supplement: Supplementary file 5 — Additional file 5: Appendix 5. LLINs tracking waybills. [file 12936_2018_2406_MOESM5_ESM.pdf]

Ministério de Saúde
Programa Nacional de Controlo da Malária
CAMPANHA DE DISTRIBUIÇÃO DE REDES MOSQUITEIRAS PARA A COBERTURA UNIVERSAL

Guia de Remessa

Nr. (Pré-numeração)

Provincia: _____________ **Distrito:** _________

Data: ____/_____/______

Enviado por: _____________________________ **Para:** ______________________________________

Local: ____________________________________ **Local:** ______________________________________

Tipo de transporte: ________________________ **Matrícula:** ____________________________

Nr	Artigo	Quantidade	Unidade	Tipo de embalagem	Quantidade de embalagens
1					
2					
3					
4					
5					
6					
7					

Comentários / observações ***(estado dos bens no acto de recepção: faltas / quebras, etc)***

Confrmação do carregamento ***(acompanhante ou motorista)***

Nome: ____

Assinatura:____________________ Data: ___/___/____

Recepção ***(agente responsável)***

Nome: ____

Assinatura:________________________ Data:___/___/____

Assinatura do quem envia: ____ **Data:** ____/____/_____

1o Branca: Quem recebe *2o Azul: Transportador* *3o Verde: Copia que volta a quem enviou* *4o Amarela: Cópia que fica no livro*
