# Supplementary material for: Planning and implementation of a countrywide campaign to deliver over 16 million long-lasting insecticidal nets in Mozambique
Source: Malar J. 2018 Jul 9;17:254. doi: 10.1186/s12936-018-2406-2 (PMC6038318; doi:10.1186/s12936-018-2406-2)
Supplement: Supplementary file 6 — Additional file 6: Appendix 6. Operational payments plan. [file 12936_2018_2406_MOESM6_ESM.pdf]

Appendix 6: Operational payments plan

Zambezia province		Predicted registrars	District with M-Pesa?		# of M-Pesa agents	Ratio registrar/Mpesa agent	Good Mpesa coverage?		District have bank?		If not, which nearest district with bank?	Distance in Km/miles	Expected amount (local currency)
	District		Yes (1)	No (1)			Yes (x)	No (x)	Yes (1)	No (1)			
1	Quelimane	703	1		372	1.9	x		1				984,200
2	Alto Molocué	720	1		48	15.0	x		1				1,008,000
3	Chinde	174		1	0	0		x		1	Mopeia	120	243,600
4	Derre	168		1	0	0		x		1	Morrumbala	90	235,200
5	Gilé	361	1		1	361.0		x		1	Alto Molocue	160	505,400
		2,126	3	2	421	5.05			2	3			2,976,400

Legend

- Up to 15 registrars per Mpesa agent - Good ratio
- 15.1 to 45.0 registrars per Mpesa agent - Moderate ratio
- 45.1 and more registrars per Mpesa agent - Bad ratio
- Without Mpesa

Zambezia province		Predicted registrars	Payment plan for registrars	Remarks	Payment plan for health staff	Remarks
	**District**					
1	Quelimane	703	Good ratio. Mpesa payment for all registrars	All recruited registrars should have cell phone	Bank transfer	Ensure that all health staff provides accurate bank account numbers, including IBAN
2	Alto Molocué	720	Good ratio. All registrars from district headquarter should be paid through Mpesa	All recruited registrars from district headquarter should have cell phone	Bank transfer	Ensure that all health staff provides accurate bank account numbers, including IBAN
3	Chinde	174	No Mpesa agent available. Payments should be made through cash on hand	Ensure money withdraw in Mopeia district	Bank transfer	Ensure that all health staff provides accurate bank account numbers, including IBAN
4	Derre	168	No Mpesa agent available. Payments should be made through cash on hand	Ensure money withdraw in Morrumbala district	Bank transfer	Ensure that all health staff provides accurate bank account numbers, including IBAN
5	Gilé	361	Bad ratio. Payments through cash on hand	Ensure money withdraw in Molocue distrcit	Bank transfer	Ensure that all health staff provides accurate bank account numbers, including IBAN
		2,126				

Page 1
