# Supplementary material for: Planning and implementation of a countrywide campaign to deliver over 16 million long-lasting insecticidal nets in Mozambique
Source: Malar J. 2018 Jul 9;17:254. doi: 10.1186/s12936-018-2406-2 (PMC6038318; doi:10.1186/s12936-018-2406-2)
Supplement: Supplementary file 7 — Additional file 7: Appendix 7. Results of the household registration rapid monitoring. [file 12936_2018_2406_MOESM7_ESM.doc]

Appendix S7: Steps and results of the household registration rapid monitoring process

**Steps of the household registration rapid monitoring process**

1. Random selection of three lots (localities within the district). The household registration was planned for seven days and the sample size for each lot was set at 140. Each monitor was responsible to survey 20 households per day. The cumulative number of surveyed households over three days was 420;
2. Monitoring began in the first lot on day two of household registration;
3. In each lot, three communities (clusters) registered the day before were randomly selected;
4. Each monitor assessed 20 households, selecting the first household randomly and then the sixth household after that. If that household was closed up or there was nobody at home, the monitor went to the next immediate house. Households that did not agree to being registered were not surveyed;
5. Each monitor collected the following information: whether the household received a sticker; whether the household received a coupon; whether the household received the correct allocation of LLINs; whether the household received accurate knowledge about the distribution point location; whether the household received accurate knowledge about the use of nets;
6. At the end of the day, the monitoring team lead summed up data for each indicator, based on the number of “fails” for the first three items (number of stickers, number of coupons and number of households with incorrect allocation of LLINs). The decision on action to be taken was then made according to the decision rules;
7. On day 3, monitors went to lot 2 and repeated the steps above;
8. On day 4, the monitors went to lot 3 and repeated the steps above. The results were then cumulative, including the number of “fails” in the three lots.

The following decision rules were adopted: “Fail” in 0 - 31 households (with 420 systematically surveyed households) means the registration is acceptable; 32 and more fails with at least 09 fails in each lot, then the whole district should have a repeat household registration.

**Results of the household registration rapid monitoring process**

Table 1 Three days result from household registration independent monitoring process in Mozambique, 2016 – 2017: fails with sticker, coupons and incorrect LLINs ascription

| Provinces | Surveyed HHs | Houses without sticker | | Houses without coupons | | Houses with incorrect LLINs ascription | |
| --- | --- | --- | --- | --- | --- | --- | --- |
|  | N | n | % | n | % | N | % |
| Cabo Delgado | 7,173 | 98 | 1.37 | 0 | 0.00 | 131 | 1.83 |
| Niassa | 6,200 | 34 | 0.55 | 33 | 0.53 | 140 | 2.26 |
| Nampula | 11,514 | 632 | 5.49 | 1,035 | 8.99 | NA | NA |
| Zambezia | 9,200 | 44 | 0.48 | 44 | 0.48 | 262 | 2.85 |
| Tete | 6,224 | 135 | 2.17 | 121 | 1.94 | 261 | 4.19 |
| Manica | 4,952 | 1 | 0.02 | 5 | 0.10 | 90 | 1.82 |
| Sofala | 5,395 | 0 | 0.00 | 21 | 0.39 | 140 | 2.59 |
| Inhambane | 5,824 | 1 | 0.02 | 1 | 0.02 | 45 | 0.77 |
| Gaza | 5,901 | 36 | 0.61 | 14 | 0.24 | 273 | 4.63 |
| Maputo Province | 2,484 | 0 | 0.00 | 13 | 0.52 | 47 | 1.89 |
| Total | 64,867 | 981 | 1.51 | 1,287 | 1.98 | 1,389 | 2.14 |

NA – Not applicable. In Nampula province, only stickers and coupons were monitored during the rapid monitoring process.

Table 2 Three days result from household registration independent monitoring process in Mozambique, 2016 – 2017: fails explaining the location of the distribution center and on how to use LLINs

| Provinces | Surveyed HHs | Households not knowing the distribution center | | Households not explained on how to use LLINs | |
| --- | --- | --- | --- | --- | --- |
|  | N | n | % | n | % |
| Cabo Delegado | 7,173 | 393 | 5.48 | 571 | 7.96 |
| Niassa | 6,200 | 328 | 5.29 | 430 | 6.94 |
| Nampula | NA | NA | NA | NA | NA |
| Zambezia | 9,200 | 596 | 6.48 | 1,025 | 11.14 |
| Tete | 6,224 | 667 | 10.72 | 927 | 14.89 |
| Manica | 4,952 | 304 | 6.14 | 748 | 15.11 |
| Sofala | 5,395 | 294 | 5.45 | 634 | 11.75 |
| Inhambane | 5,824 | 255 | 4.38 | 243 | 4.17 |
| Gaza | 5,901 | 1,052 | 17.83 | 1,426 | 24.17 |
| Maputo Province | 2,484 | 331 | 13.33 | 521 | 20.97 |
| Total | 53,353 | 4,220 | 7.91 | 6,525 | 12.23 |

NA – Not applicable. In Nampula province, only stickers and coupons were monitored during the rapid monitoring process
